# Cortico‐striato‐thalamo‐cerebellar networks of structural covariance underlying different epilepsy syndromes associated with generalized tonic–clonic seizures

**DOI:** 10.1002/hbm.25279

**Published:** 2020-12-29

**Authors:** Qiang Xu, Qirui Zhang, Fang Yang, Yifei Weng, Xinyu Xie, Jingru Hao, Rongfeng Qi, Valentina Gumenyuk, Steven M Stufflebeam, Boris C. Bernhardt, Guangming Lu, Zhiqiang Zhang

**Affiliations:** ^1^ Department of Medical Imaging, Jinling Hospital Medical school of Nanjing University Nanjing China; ^2^ College of Automation Engineering Nanjing University of Aeronautics and Astronautics Nanjing China; ^3^ Department of Neurology, Jinling Hospital Medical school of Nanjing University Nanjing China; ^4^ Athinoula A. Martinos Center for Biomedical Imaging, Department of Radiology Massachusetts General Hospital, Harvard Medical School Charlestown Massachusetts USA; ^5^ Multimodal Imaging and Connectome Analysis Laboratory, Montreal Neurological Institute and Hospital McGill University Montreal Canada; ^6^ State Key Laboratory of Analytical Chemistry for Life Science Nanjing University Nanjing China

**Keywords:** cortico‐striato‐thalamo‐cerebellar network, epilepsy, generalized tonic–clonic seizures, morhpometric MRI, structural covariance connecvity

## Abstract

Generalized tonic–clonic seizures (GTCS) are the severest and most remarkable clinical expressions of human epilepsy. Cortical, subcortical, and cerebellar structures, organized with different network patterns, underlying the pathophysiological substrates of genetic associated epilepsy with GTCS (GE‐GTCS) and focal epilepsy associated with focal to bilateral tonic–clonic seizure (FE‐FBTS). Structural covariance analysis can delineate the features of epilepsy network related with long‐term effects from seizure. Morphometric MRI data of 111 patients with GE‐GTCS, 111 patients with FE‐FBTS and 111 healthy controls were studied. Cortico‐striato‐thalao‐cerebellar networks of structural covariance within the gray matter were constructed using a Winner‐take‐all strategy with five cortical parcellations. Comparisons of structural covariance networks were conducted using permutation tests, and module effects of disease duration on networks were conducted using GLM model. Both patient groups showed increased connectivity of structural covariance relative to controls, mainly within the striatum and thalamus, and mostly correlated with the frontal, motor, and somatosensory cortices. Connectivity changes increased as a function of epilepsy durations. FE‐FBTS showed more intensive and extensive gray matter changes with volumetric loss and connectivity increment than GE‐GTCS. Our findings implicated cortico‐striato‐thalamo‐cerebellar network changes at a large temporal scale in GTCS, with FE‐FBTS showing more severe network disruption. The study contributed novel imaging evidence for understanding the different epilepsy syndromes associated with generalized seizures.

## Graphical abstract

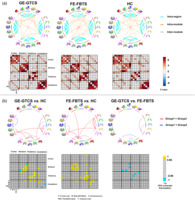

## 1 INTRODUCTION

Generalized tonic–clonic seizures (GTCS), traditionally termed grand mal seizures, are the severest and most remarkable clinical expressions of human epilepsy, which typically presents symptoms of muscle rigidity, violent muscle contractions of entire body and complete loss of consciousness, and can cause injury and even death (Blumenfeld & Taylor, 2003). GTCS are often seen in genetic generalized epilepsy (GE‐GTCS) syndromes (also referred to as idiopathic generalized epilepsy) (Scheffer et al., 2017). They can also be observed secondary to partial seizure onset in focal epilepsy syndromes, and are referred as focal to bilateral tonic–clonic seizure (FE‐FBTS) (formerly called secondarily GTCS) (Fisher, 2017). Although both syndromes show GTCS, GE‐GTCS immediately presents generalized symptoms at the beginning of seizure onset, while FE‐FBTS presents generalized symptoms secondary to an initial focal onset. Different diagnosis between these two epilepsy types is clinically significant for establishing pharmacotherapeutic schemes (Shorvon, Bermejo, Gibbs, Huberfeld, & Kälviäinen, 2018).

These two types of GTCS have different neural pathophysiological mechanisms (Gloor, 1968). According to the epilepsy network theory, in GE‐GTCS, seizures originate from the midbrain and thalamus, and rapidly engage bilaterally distributed brain networks; while in FE‐FBTS, seizures unilaterally originate from a cortical focus, initially propagates to the thalamus, and subsequently to bilaterally distributed brain regions via cortico‐thalamic networks (Berg et al., 2010; Blumenfeld et al., 2009; Pegg, Taylor, Keller, & Mohanraj, 2020). Among the epilepsy networks in both syndromes of GTCS, besides to the essential structures of the cerebral cortex and thalamus, the striatum and cerebellum are also playing important roles for propagation, termination and modulation of seizure activity (see review by [Norden & Blumenfeld, 2002]). A growing body of imaging literatures has demonstrated structural and functional abnormalities in these regions in the syndromes (Blumenfeld & Taylor, 2003; Ciumas & Savic, 2006; Szabó et al., 2006; Zhou et al., 2015). Especially, network‐sensitive imaging has delineated epilepsy network in various modalities. FMRI studies have demonstrated generalized spike and wave‐discharges related activation in thalamus and deactivation in the default mode network, and widespread changes of intrinsic connectivity networks associated with disease duration in GE‐GTCS (Aghakhani et al., 2004; Gotman et al., 2005; Moeller et al., 2008). Previous studies showed dynamic perfusion changed from cerebral cortex to thalamus, basal ganglia, and ended to the cerebellum, midbrain and basal ganglia in FE‐FBTS (Blumenfeld et al., 2009; Englot et al., 2010). Basal ganglia inhibition and thalamus synchronization were the key factors of presence and effective control of focal to bilateral tonic–clonic seizures (He et al., 2020). Our previous found that GE‐GTCS showed a more constrained network embedding of thalamus, and thalamocortical network imbalance were related to future drug response (Wang et al., 2019). In addition, we observed functional connectivity alteration of cortico‐thalamic network projected from five specific cortical lobes in GE‐GTCS, suggesting the corticothalamic network underlying the pathophysiological mechanism of GE‐GTCS (Ji et al., 2015).

In comparison with functional measures on transient changes of brain activity, morphometric analysis can provide a relative stable estimate of brain organization and tap into accumulating injury effect over long time windows(Alexander‐Bloch, Giedd, & Bullmore, 2013; Evans, 2013; Liao et al., 2013; Zhang et al., 2017). Thus, structural covariance network (SCN), constructed by morphometric correlation analysis, can measure synchronized structural alterations within a network undergoing common pathological processes (Alexander‐Bloch et al., 2013; Evans, 2013; Mechelli, Friston, Frackowiak, & Price, 2005; Yun et al., 2020). SCN can recapitulate the topological patterns of intrinsic functional networks measured by functional and anatomic network by BOLD‐fMRI and DTI in physiological or disease states (Gong, He, Chen, & Evans, 2012; Keller et al., 2014; Seeley, Crawford, Zhou, Miller, & Greicius, 2009). Boris and colleagues have delineated the thalamic‐cortical network of structural covariance in GE‐GTCS, and elaborated the relationship with long‐duration impairment effect from disease (Bernhardt et al., 2009). Our previous work demonstrated large‐scale graph property of epileptic networks in GE‐GTCS using SCN, and further comparing with the intrinsic connectivity network domain, indicating SCN could reflect shared long‐term trophic mechanisms within functionally synchronous systems (Liao et al., 2013). However, so far, no study has addressed the precise network patterns of structural covariance among cerebral cortex, thalamus, striatum, and cerebellum. Especially, we are not yet clear about the difference of network features underlying the two types of GTCSs.

In this work, we adopted SCN approach to map structural network organization in GE‐GTCS and FE‐FBTS. We adopted prior paradigms to stratify networks relative to large‐scale communities (Buckner, Krienen, Castellanos, Diaz, & Yeo, 2011; Ji et al., 2015; Wang et al., 2019; D. Zhang et al., 2008). In addition to studying effects in patient cohorts relative to controls, we assessed duration effects. This work would provide imaging evidence for shaping the patterns of epilepsy networks, and also gave insights into the pathophysiological mechanism of brain structural impairments in the two GTCS syndromes.

## 2 MATERIALS AND METHODS

### 2.1 Participants

We studied 111 patients with GE‐GTCS and 111 age‐ and sex‐matched FE‐FBTS patients, recruited from 2009 to 2017 in Jinling hospital (detailed in Table 1). All patients were diagnosed according to seizure symptoms, scalp‐EEG and therapeutic responses, re‐checked and cross validated by two experienced neurologists (F. Y. and G. C.) according to the criteria of 2017 International League Against Epilepsy classification scheme (Scheffer et al., 2017). For GE‐GTCS, the patients had: (a) Typical manifestation of generalized tonic–clonic seizures, including tonic extension of the limbs followed by a clonic phase of rhythmic jerking of the extremities and loss of consciousness during seizures without precursory symptoms of partial epilepsy and aura; (b) presence of GSWD on the video‐electroencephalogram; and no other epilepsy associated etiology such as trauma, tumor, intracranial infection. The exclusive criteria included: (a) History of epilepsy associated etiology such as trauma, tumor, and intracranial infection. (b) Mixed type of other genetic epilepsy, such as absence seizures and juvenile myoclonic epilepsy, age younger than 18 years of older than 50 years. Seventy‐two patients had been taking anti‐epileptic medications on the time of scan, including: Sodium Valproate (41 cases), Carbamazepine (21 cases), Lamorgine (10 cases), Topiramate (7 cases), Levetriaracetam(6 cases), phenobarbita (4 cases), Clonazepam (3 cases), Oxcarbazepine (1 cases).

**TABLE 1:** Demographic and clinical information of subjects

	Age	Gender	Epilepsy duration
	(yo, mean ± *SEM*)	(male/female)	(mths, mean ± *SEM*)
GE‐GTCS	25.82 ± 0.74	71/40	82.85 ± 8.926
FE‐FBTS	25.74 ± 0.76	71/40	82.99 ± 8.62
HCs	26.26 ± 0.69	71/40	–
Statistic	F = 0.147, *p* = .863^a^	Chi‐square = 0.000, *p* = 1.000 ^b^	t = 0.575, *p* = .566^c^

*Note:* a, One‐way ANOVA test; b, Chi‐square test; c, Two‐sample *t* test.

For FE‐FBTS, the patients presented: (a) typical symptoms of focal frontal seizures, such as head and eye movement to one side, abnormal body posturing or difficulty speaking prior to GTCS onset, and (b) focal frontal epileptic discharges on EEG. The exclusive criteria included: (a) Visible focal abnormalities on structural MRI imaging detected by an experienced radiologist(ZZ). (b) Ages younger than 18 years or older than 50 years. Eight‐six patients had been taking anti‐epileptic medications including: Sodium Valproate (32 cases), Carbamazepine (25 cases), Topiramate (18 cases), Oxcarbazepine (14 cases), Phenobarbital (10 cases), Phenytoin (9 cases), Lamotrigine (6 cases), Levetiracetam (3 cases), Clonazepam (2 cases). All the patients had no abnormality on structural MR imaging.

Moreover, 111 age‐ and gender‐matched healthy controls (HCs) were recruited from the staff of Jinling Hospital. All participants were right‐handedness. This study was approved by the Medical Ethics Committee in Jinling Hospital, Nanjing University School of Medicine. Written informed consents were obtained from all the participants.

### 2.2 MRI acquisition and data processing

All participants were scanned in 3 T MRI scanner (Siemens Trio, Germany). High‐resolution T1‐weighted anatomical images were acquired in the sagittal orientation using a magnetization‐prepared rapid gradient‐echo sequence with following parameters: TR = 2,300 ms, TE = 2.98 ms, Flip Angle = 9°, FOV = 25.6 × 25.6 cm^2^, Acquisition Matrix = 256 × 256, slice thickness = 1 mm, 176 slices without interslice gap.

Voxel‐based morphometry (VBM) analysis on high‐resolution T1‐weighted images were performed using CAT12 (http://www.neuro.uni-jena.de/cat/) implemented in SPM12 (http://www.fil.ion.ucl.ac.uk/spm). The images of each subject were transformed into standard MNI space with a 12‐parameter affine‐only non‐linear transformation, and resampled to 1.5 × 1.5 × 1.5 mm^3^. Then the images were segmented into three tissue classes representing gray matter, white matter, and cerebrospinal fluid. The resultant probabilistic gray matter maps were further smoothed with an 8 mm FWHM isotropic Gaussian kernel used for the following structural covariance network analysis.

### 2.3 Mapping cortico‐striato‐thalamo‐cerebellar network of structural covariance

**FIGURE 1:**
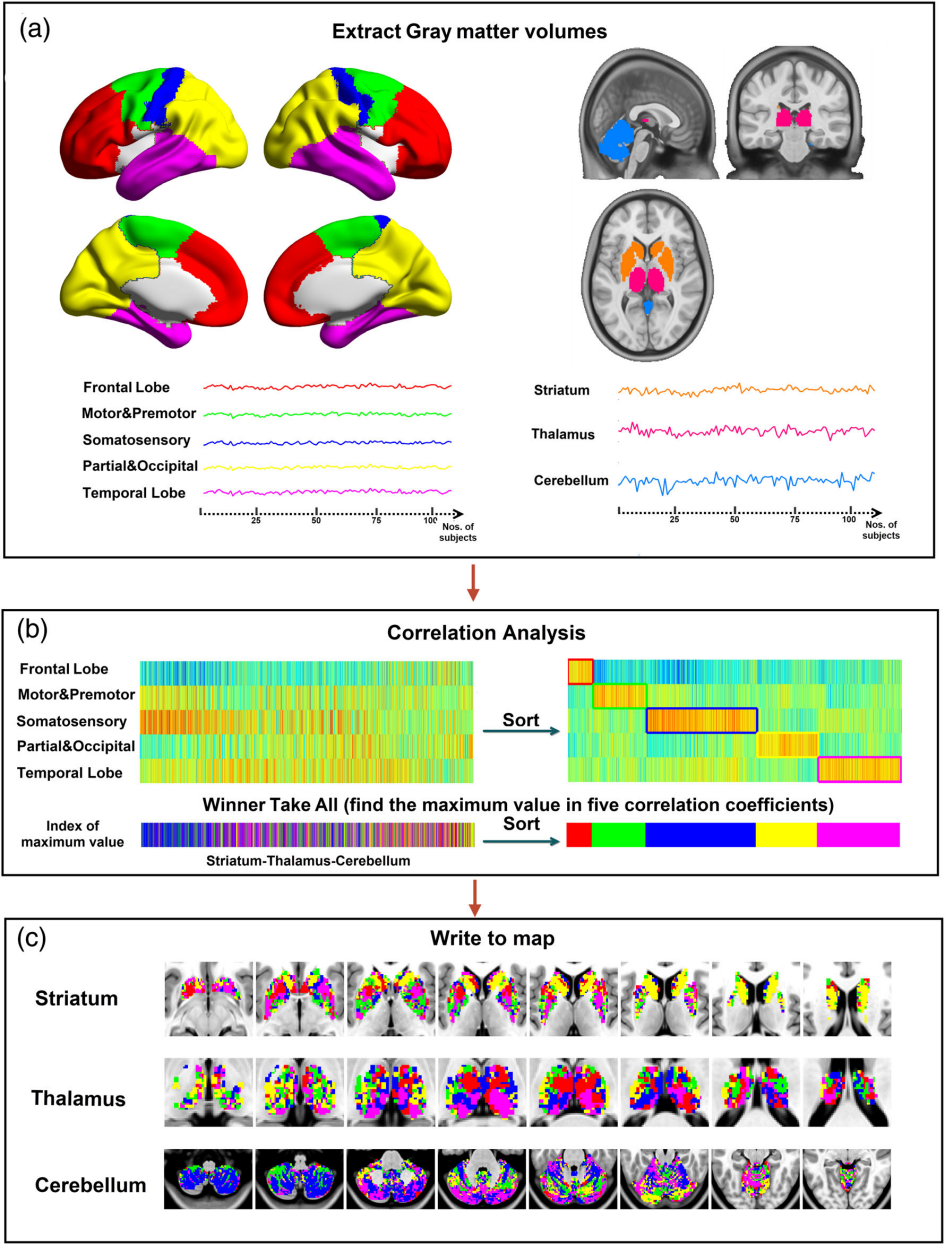
Workflow of mapping cortico‐striato, ‐thalamo, and ‐cerebellar connectivity of structural covariance using winner‐take‐all analysis. (a) Five cortical parcellations as seeding regions and subcortical structures as targeting regions. (b) Correlation analysis between signals of voxles in each subcortical structures and averaged signals in each cortical parcellation. The voxel in the subcortical structure was labeled according to the cortical parcellation with the highest correlation coefficient. (c) Maps of cortical–subcortical‐cerebellar networks. Labeled Colors. Red: Frontal lobe; Green: Motor & Premotor; Blue: Somatosensory, Yellow: Partial & Occipital; Violet: Temporal lobe. Orange: striatum; Claret‐colored: Thalamus; Indigo: cerebellum

In line with the previous works (Ji et al., 2015; Zhang et al., 2008), we employed a cortical parcellation by dividing bilateral hemispheres into five no‐overlapping lobes based on automated anatomic labeling template: (a) Frontal lobe, (b) Motor/premotor lobe, (c) Somatosensory lobe, (d) Parietal/occipital lobe, and (e) Temporal lobe. The cortical parcellations were defined by taking into account the known connectivity between the thalamus and cortex (Behrens et al., 2003). In each group of patients and HCs, the averaged gray‐matter volume (GMV) values in the cortical parcellation were extracted from each subject; then calculated the structural covariance between the averaged volume sequence within each lobe and each voxel of the thalamus, striatum, and cerebellum respectively by using partial correlation. The masks of thalamus, striatum, and cerebellum were also acquired from automated anatomic labeling template. Structural covariance computations for a given lobe involved setting the GMV of other lobes and total intracranial volumes (TIV) were set as covariates. Each voxel of the striatum, thalamus and cerebellum was labeled according to the cortical lobe with the highest correlation coefficient (i.e., “winner take all”) (Ji et al., 2015; Zhang et al., 2008), respectively. Thus, the striatum, thalamus, and cerebellum could be separated into five subregions in each group (see Figure 1).

### 2.4 VBM analysis and relevancies with progressive factors in epilepsy

Firstly, GMV data of three participant groups were compared using analysis of variance implemented in SPM12 (*p* < .05, Gaussian random field theory correction, with voxel level *p* < .01 and cluster level *p* < .05, by using DPABI toolbox, http://rfmri.net/dpabi), for mapping the different patterns of GMV alterations in the patients. Subsequently, voxel‐wise Pearson correlation analysis was performed between GMV data and epilepsy duration in each patient group. Total intracranial volume, sex, and age were added as covariates of no interest (Hong et al., 2019; Zhang et al., 2017). Moreover, a linear‐interaction model analysis was used to investigate the differences of correlation coefficient between two patient groups using SurfStat for Matlab (http://www.math.mcgill.ca/keith/surfstat/#ICBMagain) (Bernhardt et al., 2009; Lerch et al., 2006; Worsley, Taylor, Carbonell, Chung, & Evans, 2009).

### 2.5 Cortico‐subcortical network analysis

Firstly, the Winner‐Take‐All (WTA) maps of cortico‐striatal, cortico‐thalamic, and cortico‐cerebellar networks of structural covariance were compared respectively among three groups using permutation tests (5,000 permutation). During each pairwise permutation test, the labels of two groups were rearranged randomly (Liao et al., 2013; Teipel et al., 2016).

We also carried out permutation tests (5,000 permutations) for the cortico‐striato‐thalamo‐cerebellar network. For constructing the cortico‐striato‐thalamo‐cerebellar network, the signals (GMV values in each group) were extracted and averaged from the five cortical parcellations and the subregions showing covariance with each parcellation in the striatum, thalamus, and cerebellum. Partial correlation was used for constructing the network with 20 × 20 matrix in each group. The comparison results of network were corrected by False Positive Adjustment (FPA) (Fornito, Yoon, Zalesky, Bullmore, & Carter, 2011).

### 2.6 Associations to disease progression

In line with previous works (Bernhardt, Klimecki, Leiberg, & Singer, 2014; Sharda, Khundrakpam, Evans, & Singh, 2016; Valk et al., 2017), we adopted a fitted‐linear model analysis using SurfStat Toolbox for assessing the relationship between structural covariance network configurations and clinical variable of epilepsy duration. Linear models estimated not only the main effects of GMV in seed regions and epilepsy duration, but also the parametric interaction between them. The model fitted at the GMV in target seed region was:

$${\mathrm{GM}}_{\text{target}}=\beta_{1}\times {\mathrm{GM}}_{\text{seed}}+\beta_{2}\times \text{Duration}+\beta_{3}\times {\mathrm{GM}}_{\text{seed}}\times \text{Duration}+\beta_{4}\times \mathrm{TIV}$$

Where × indicated an interaction between terms, and TIV was the covariate of no interest. The significance of β_3_ could represent the power of the modulation effect on the structural covariance connectivity between seed ROI and target ROI.

To assess duration effects on cortico‐subcortical covariance networks, we expanded the fitted linear model, by including main effects of GMV in seed regions, epilepsy duration, the interaction effects between GMV in seed regions and epilepsy duration, the interaction effects between epilepsy duration and group, the interaction effects between GMV in seed regions and epilepsy duration, the triple interaction effects between GMV in seed regions, group and duration. The model fitted at the GMV in target seed region was:

$${\mathrm{GM}}_{\text{target}}=\beta_{1}\times \text{Group}+\beta_{2}\times {\mathrm{GM}}_{\text{seed}}+\beta_{3}\times \text{Duration}+\beta_{4}\times \text{Group}\times {\mathrm{GM}}_{\text{seed}}+\beta_{5}\times \text{Group}\times \text{Duration}+\beta_{6}\times {\mathrm{GM}}_{\text{seed}}\times \text{Duration}+\beta_{7}\times \text{Group}\times {\mathrm{GM}}_{\text{seed}}\times \text{Duration}+\beta_{8}\times \mathrm{TIV}$$

Where × indicated an interaction between terms, and TIV was the covariate of no interest. The significance of β_7_ could represent the differences of the modulation effect on the structural covariance connectivity between seeding region and target region between GE‐GTCS and FE‐FBTS. Here we only compared the differences within the same sub‐network (i.e., the network combined by frontal lobe, and subregions of striatum, thalamus, and cerebellum, which were marked by frontal lobe).

## 3 RESULTS

There was no difference in age and gender among GE‐GTCS, FE‐FBTS, and healthy controls, either no difference of disease durations between GE‐GTCS and FE‐FBTS (details in Table 1). No proportion difference of medications was found between GE‐GTCS and FE‐FBTS.

### 3.1 VBM analysis and relevancies with epilepsy duration

**FIGURE 2:**
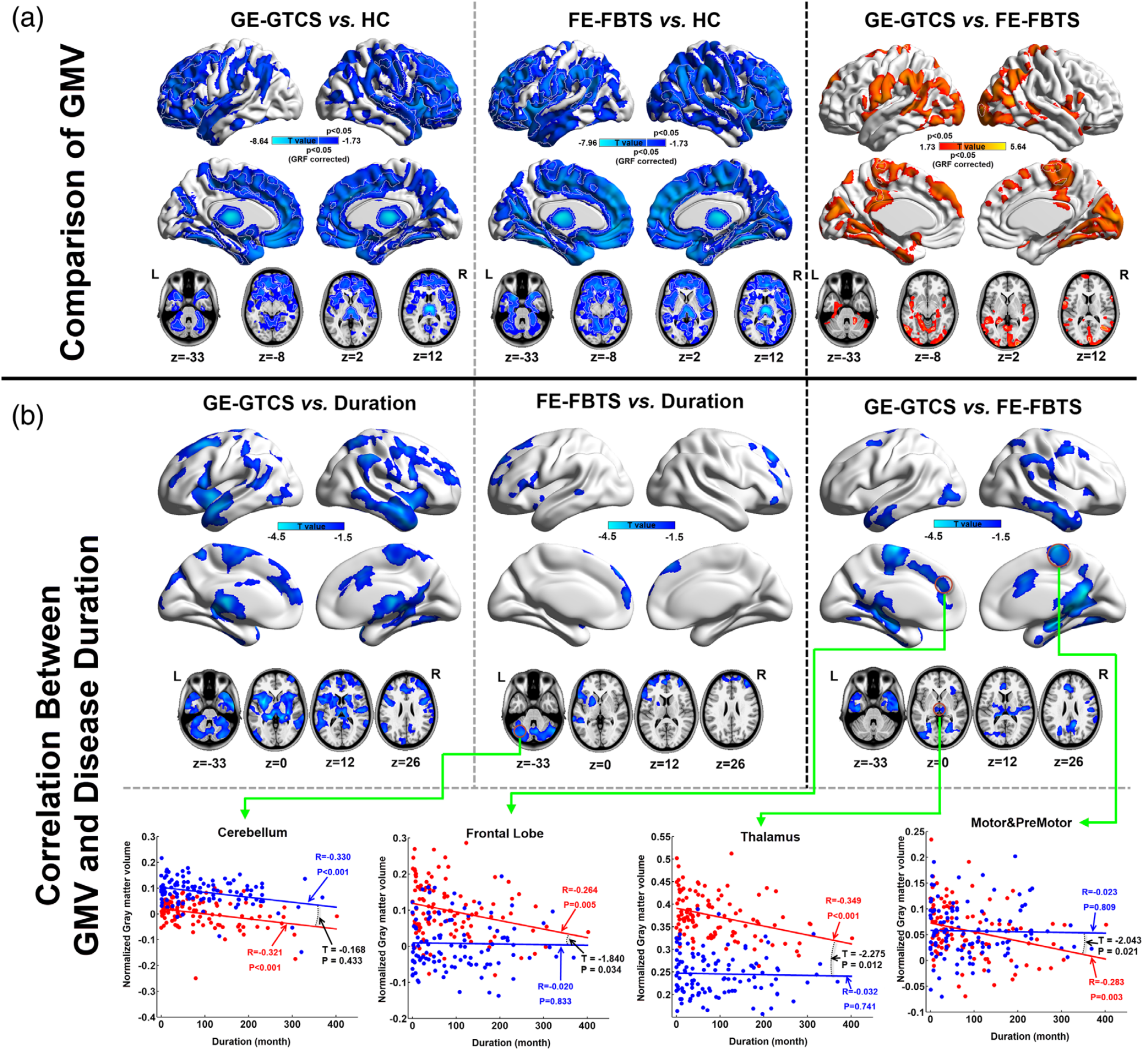
VBM analysis and relevancies with Progressive Factors in epilepsy. (a) Group comparisons of Gray matter volume among three participant groups. Left: Comparison between GE‐GTCS and HC. Middle: Comparison between FE‐FBTS and HC. Right: Comparison between GE‐GTCS and FE‐FETS. (b) Correlation analyses between GMV and epilepsy duration. Upper level. Left: Voxel‐based correlation between GMV and epilepsy duration in GE‐GTCS. Middle: Voxel‐based correlation between GMV and epilepsy duration in FE‐FBTS. Right: Comparison of relationship between GMV and epilepsy duration between GE‐GTCS and FE‐FBTS using linear‐interaction analysis. Lower level. Scatter plots for showing the correlation between GMV and epilepsy duration in several representative regions with region‐of‐interest wise

Compared to the HCs, both the patient groups showed widespread GMV decrease within the bilateral cortical structures, head of caudate, thalamus, and cerebellum. The cortical GMV decreases were predominantly located at the anterior part of the brain. The patients with FE‐FBTS presented more intensive and extensive GMV decrease in the cortical structures relative to the GE‐GTCS (see Figure 2 *top panel*). Both two patient groups showed negative correlation between GMV and epilepsy duration in the cortical structures, subcortical nuclei, and cerebellum (see Figure 2 *middle panel*). However, the GE‐GTCS patients presented stronger negative correlation than the FE‐FBTS, especially in the mesial frontal lobe, sensorimotor cortex and thalamus (see Figure 2 *bottom panel*).

### 3.2 Comparisons of cortico‐striato‐thalamo‐cerebellar networks

#### 3.2.1 Map comparisons

**FIGURE 3:**
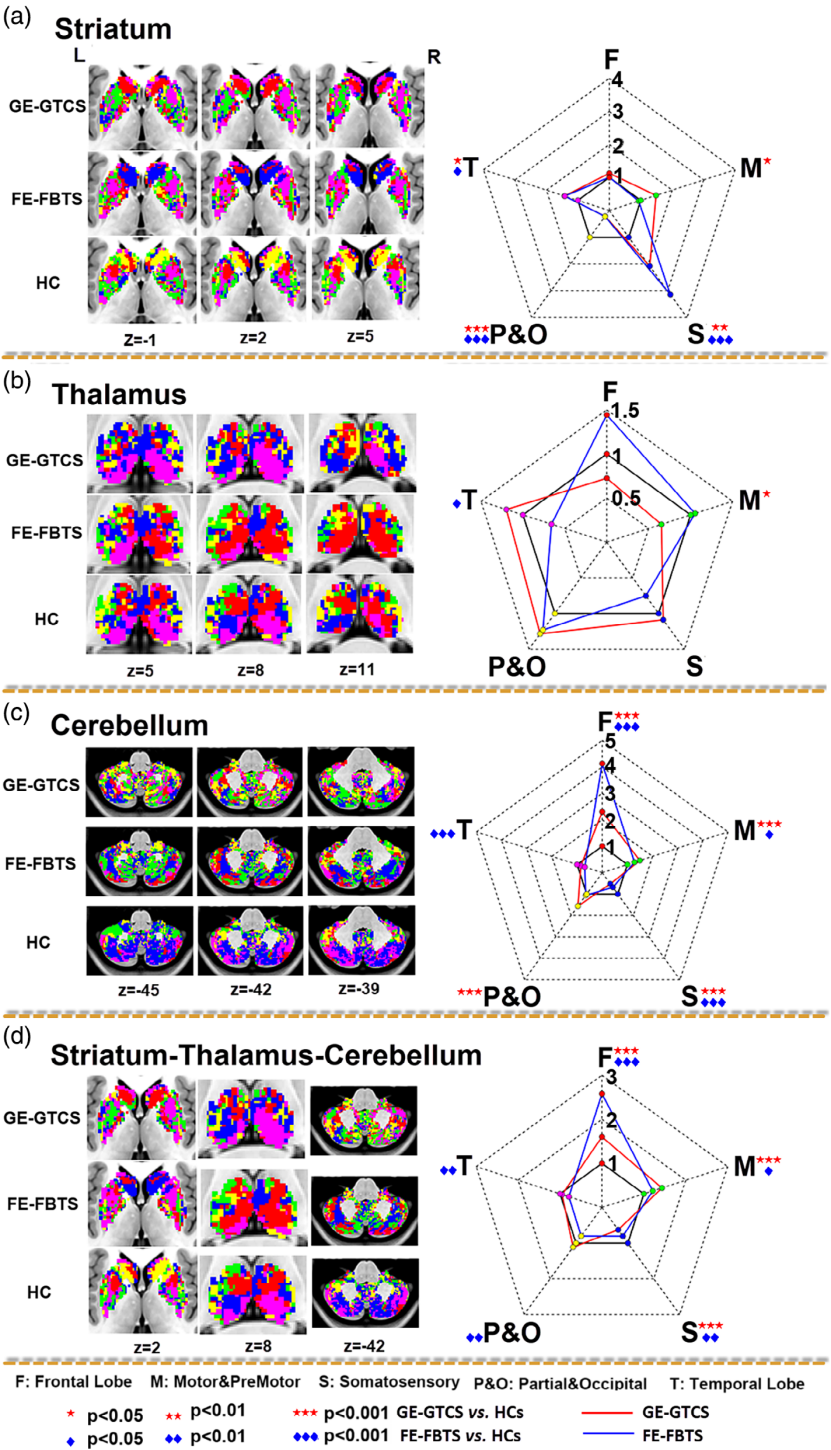
Maps of cortico‐striatal connectivity (a), cortico‐thalamic connectivity (b), cortico‐cerebellar connectivity (c), and combining striatum‐thalamus‐cerebellum connectivity (d) using winner‐take‐all. (a) Left column: Maps of connectivity in each structure and each group. Red: voxels showing connectivity to the frontal lobe, Green: voxels showing connectivity to the motor/premotor lobe, Blue: voxels showing connectivity to the somatosensory cortex, Yellow: voxels showing connectivity to the parietal/occipital lobe, and violet: voxels showing connectivity to the temporal lobe. (b) Right column: Group comparisons of SCN maps. In radar image, the scale in each axis represents relative number of voxels showing connectivity to the corresponding cortical structures, the relative number was standardized by dividing the voxel number of healthy controls

Number of voxels showing covariance connectivity was used in the map comparisons between patients and healthy controls. For the cortical‐striatum networks, both patient groups showed more somatosensory cortex‐striatum covariance, but less parietal/occipital cortex‐striatum covariance relative to healthy controls (see Figure 3 *panel A* and Table 2 *panel Striatum*). For the cortical‐thalamus networks, the GE‐GTCS showed more temporal cortex‐thalamus covariance, and the FE‐FBTS showed more frontal cortex‐thalamus covariance and motor cortex‐thalamus covariance relative to healthy controls (see Figure 3 *panel B* and Table 2 *panel Thalamus*). For the cortical‐cerebellum networks, both the patient groups showed more frontal cortex‐cerebellum covariance (see Figure 3 *panel C* and Table 2 *panel Cerebellum*). In addition, for the comparison of overall maps, both the patient groups showed more covariance in the networks of frontal cortex (see Figure 3 *panel D* and Table 2 *panel Straitum‐Thalamus‐Cerebellum*).

**TABLE 2:** Pairwise comparisons of connected voxel numbers of Cortex ROIs in striatum, thalamus, cerebellum among three groups

	GE‐GTCS vs. HC			FE‐FBTS vs. HC			GE‐GTCS vs. FE‐FBTS		
Seed ROIs	GE‐GTCS	HC	*p* value	FE‐FBTS	HC	*p* value	GE‐GTCS	FE‐FBTS	*p* value
Striatum									
Increased									
Motor and PreMotor	2,273	1,530	**.020**	–	–	–	2,273	1,429	**.011**
Somatosensory	2006	965	**.008**	3,030	965	**.000**	–	–	–
Temporal lobe	2,933	2061	**.027**	2,919	2061	**.029**	–	–	–
Decreased									
Somatosensory	–	–	–	–	–	–	2006	3,030	**.025**
Parietal and occipital	791	3,638	**.000**	806	3,638	**.000**	–	–	–
Thalamus									
Increased									
Temporal lobe	–	–	–	–	–	–	1,298	715	**.003**
Decreased									
Frontal lobe	–	–	–	–	–	–	714	1,421	**.004**
Motor &PreMotor	477	732	**.034**	–	–	–	477	771	**.006**
Temporal lobe	–	–	–	715	1,084	**.037**	–	–	–
Cerebellum									
Increased									
Frontal lobe	5,119	2,224	**.000**	9,179	2,224	**.000**	–	–	–
Motor and PreMotor	11,550	7,730	**.000**	9,916	7,730	**.015**	–	–	–
Parietal and occipital	10,915	6,985	**.000**	–	–	–	10,915	7,388	**.000**
Temporal lobe	–	–	–	–	–	–	10,434	8,533	**.013**
Decreased									
Frontal lobe	–	–	–	–	–	–	5,119	9,179	**.000**
Somatosensory	9,638	18,431	**.000**	12,640	18,431	**.000**	9,638	12,640	**.004**
Temporal lobe	–	–	–	8,533	12,286	**.001**	–	–	–
Striatum‐thalamus‐cerebellum									
Increased									
Frontal lobe	7,501	4,686	**.000**	12,087	4,686	**.000**	–	–	–
Motor and PreMotor	14,300	9,992	**.000**	12,116	9,992	**.026**	14,300	12,116	**.026**
Parietal and occipital	–	–	–	–	–	–	12,656	9,104	**.000**
Temporal lobe	–	–	–	–	–	–	14,665	12,167	**.008**
Decreased									
Frontal lobe	–	–	–	–	–	–	7,501	12,087	**.000**
Somatosensory	12,872	20,523	**.000**	16,520	20,523	**.007**	12,872	16,520	**.002**
Parietal and occipital	–	–	–	9,104	11,362	**.004**	–	–	–
Temporal lobe	–	–	–	12,167	15,431	**.005**	–	–	–

### 3.3 Cortico‐striato‐thalamo‐cerebellar network comparisons

**FIGURE 4:**
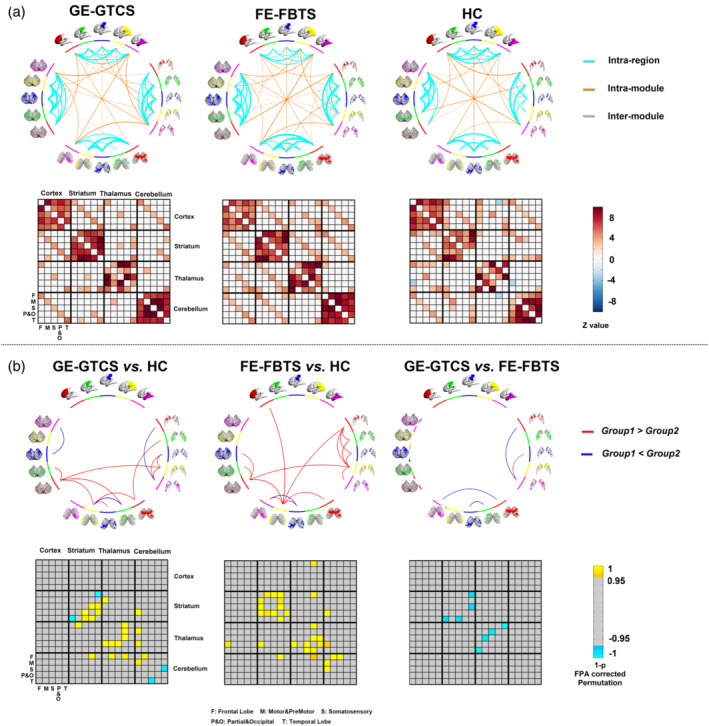
Cortico‐striato‐thalamo‐cerebellar network of structural covariance in three groups. Cortico‐striato‐thalamo‐cerebellar network pattern in each subject group. Group comparisons of cortico‐striato‐thalamo‐cerebellar networks among three groups

Generally, we first found prominent covariance connectivity within each structure of striatum, thalamus, and cerebellum. Secondly, strong covariance connectivity was also kept within the subregions with the same seeding lobe across different structures (see Figure 4 *panel A*).

Compared with the healthy controls, both patient groups showed mainly strengthened covariance connectivity, mainly in the structures of striatum and thalamus. For both patient groups, the changed covariances were mainly correlated with the subregions of the frontal and parietal lobes network belongings. For the comparison between patient groups, we found the GE‐GTCS showed weaker covariance mainly in the striatum and thalamus relative to the FE‐FBTS. Moreover, the different covariance connectivity between disease groups were mostly correlated with the subregions of the frontal lobe, temporal lobe, and somatosensory lobe network belongings (see Figure 4 *panel B* and Table 3).

**TABLE 3:** Pairwise comparison results of cortico‐ striato‐thalamo‐cerebellar covariance networks among three groups

Increased covariance connectivity		GE‐GTCS vs. FE‐FBTS	GE‐GTCS vs. HC	FE‐FBTS vs. HC
***Within striatum***		*p* value	*p* value	*p* value
Somatosensory(striatum)	Temporal lobe(striatum)	.001	.003	–
Somatosensory(striatum)	Parietal and Occipital(striatum)	–	<.001	<.001
Parietal and Occipital(striatum)	Temporal lobe(striatum)	–	<.001	.002
Frontal lobe(striatum)	Motor and Premoto(striatum)	–	–	.004
Frontal lobe(striatum)	Somatosensory(striatum)	–	–	<.001
Frontal lobe(striatum)	Parietal and Occipital(striatum)	–	–	<.001
Motor and Premoto(striatum)	Parietal and Occipital(striatum)	–	–	.005
***Within thalamus***				
Frontal lobe(thalamus)	Parietal and Occipital(thalamus)	–	<.001	<.001
Somatosensory(thalamus)	Parietal and Occipital(thalamus)	–	<.001	<.001
Parietal and Occipital(thalamus)	Temporal lobe(thalamus)	–	–	.003
***Within cerebellum***				
Frontal lobe(cerebellum)	Motor and Premoto(cerebellum)	–	.004	<.001
Frontal lobe(cerebellum)	Somatosensory(cerebellum)	–	–	<0.001
***Between cortex and thalamus***				
Frontal lobe(cortex)	Parietal and Occipital(thalamus)	–	–	.005
***Between striatum and thalamus***				
Frontal lobe(striatum)	Parietal and Occipital(thalamus)	–	–	.002
Motor and Premoto(striatum)	Frontal lobe(thalamus)	–	<.001	–
***Between striatum and cerebellum***				
Frontal lobe(striatum)	Parietal and Occipital(cerebellum)	–	<.001	<.001
***Between thalamus and cerebellum***				
Motor and Premoto(thalamus)	Frontal lobe(cerebellum)	–	.002	–
Parietal and Occipital(thalamus)	Frontal lobe(cerebellum)	–	.003	.01
**Decreased covariance connectivity**		GE‐GTCS vs. FE‐FBTS	GE‐GTCS vs. HC	FE‐FBTS vs. HC
***Within striatum***		*p* value	*p* value	*p* value
Frontal lobe(striatum)	Temporal lobe(striatum)	.007	<.001	–
***Within thalamus***				
Frontal lobe(thalamus)	Temporal lobe(thalamus)	.001	–	–
Motor and premoto(thalamus)	Somatosensory(thalamus)	.004	–	–
Motor and Premoto(thalamus)	Parietal and Occipital(thalamus)	–	.006	–
Somatosensory(thalamus)	Temporal lobe(thalamus)	–	–	<.001
***Within cerebellum***				
Somatosensory(cerebellum)	Temporal lobe(cerebellum)	–	.001	–

*Note:* In the first and second columns of the table, the index used the form of Seed ROI (Target Region), for example, Somatosensory(Cerebellum) represented the subregion of Cerebellum whose seed region was Somatosensory lobe.

### 3.4 Modulation effects of disease duration to cortico‐striato‐thalamo‐cerebellar network

**FIGURE 5:**
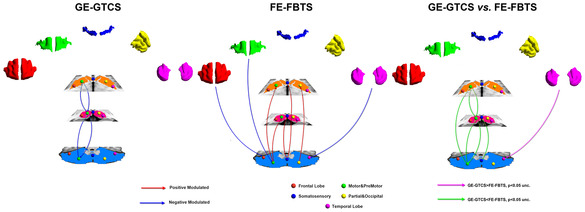
Modulation effects of epilepsy duration on the cortico‐striato‐thalamo‐cerebellar connectivity of structural covariance in patient groups. (a) Modulation effects of disease duration to network in GE‐GTCS. The covariances of Striato‐thalamo and thalamo‐cerebellar of motor cortex are negatively affected. (b) Modulation effects of disease duration to network in FE‐FBTS. The covariances of cortical structures (frontal, temporal and motor lobe) to cerebellum are negatively affected, and those of striato‐cerebellar and thalamo‐cerebellar of motor and somatosensory are positively affected. (c) Differences of modulation effects of disease duration to network between GE‐GTCS and FE‐FBTS. Relative to the FE‐FBTS, the GE‐GTCS show more negative modulation effect of disease duration on convariances of the striato‐thalamo‐cerebellar and cerebellar‐striatal circles of motor and somatosensory

For the GE‐GTCS, epilepsy duration presented negative effects reciprocally on the striato‐thalamo and thalamo‐cerebellar covariance circles of the motor related subnetwork (see Figure 5 *left panel*, Table 4). For the FE‐FBTS, epilepsy duration presented negative effects on the covariances from the cortical structures (frontal, temporal lobes and motor lobe) to cerebellum, and positive effect on the striato‐cerebellar and thalamo‐cerebellar covariances correlated with motor and somatosensory network belongings (see Figure 5 *middle panel*, Table 4). For the comparison between patient groups, the disease duration posed more negative effect on the covariances of the striato‐thalamo‐cerebellar and cerebellar‐striatal circles covariance correlated with motor and somatosensory network belongings in GE‐GTCS relative to the FE‐FBTS (see Figure 5 *right panel* and Table 4).

**TABLE 4:** Modulation effects of duration of disease to cortico‐striato‐thalamo‐cerebellar connectivity of structural covariance within and between GE‐GTCS and FE‐FBTS

Seed ROI	Target ROI	GE‐GTCS vs. FE‐FBTS		GE‐GTCS		FE‐FBTS	
		T	*p* value	T	*p* value	T	*p* value
***Frontal lobe related subnetwork***							
Cortex	Cerebellum	1.120	.132	−0.287	.388	**−2.284**	.012*
***Motor and premotor related subnetwork***							
Cortex	Cerebellum	0.320	.375	−0.940	.175	**−1.999**	.024*
Striatum	Thalamus	**−1.870**	.031*	**−1.983**	.025*	0.591	.278
Thalamus	Striatum	**−1.943**	.027*	**−2.619**	.005*	0.767	.222
Thalamus	Cerebellum	**−2.172**	.015*	**−2.979**	.002*	0.580	.282
Cerebellum	Striatum	**−2.076**	.020*	−0.616	.270	**2.090**	.020*
Cerebellum	Thalamus	**−2.966**	.002*	**−1.930**	.028*	**2.158**	.017*
***Somatosensory related subnetwork***							
Cerebellum	Striatum	−1.580	.058	−0.188	.426	**2.339**	.011*
Cerebellum	Thalamus	**−2.334**	.010*	−1.086	.140	**2.273**	.013*
***Partial and Occipital Lobe related subnetwork***							
Cortex	Cerebellum	1.297	.098	−0.865	.195	**−2.314**	.011*
Cerebellum	Striatum	−0.723	.235	−0.902	.336	**1.659**	.050*
***Temporal lobe related subnetwork***							
Cortex	Cerebellum	**1.772**	.039*	0.558	.289	**−1.816**	.036*
Cerebellum	Cortex	1.406	.081	0.255	.400	**−1.721**	**.040** *

## 4 DISCUSSION

By adopting a winner‐take‐all strategy (Ji et al., 2015; Zhang et al., 2008), we delineated the cortico‐striato‐thalamo‐cerebellar networks of structural covariance in two types of epilepsy with generalized seizures. We found that two epilepsies both presented disease duration‐associated GMV losses within widespread structures including the cortex (predominantly in the forebrain), striatum, thalamus, and cerebellum. Second, both patient groups showed strengthened connectivity of structural covariance mainly within the striatum and thalamus, and mostly correlated with the frontal, motor, and somatosensory cortices. This covariance connectivity changes were affected by the epilepsy durations. Finally, we found the FE‐FBTS showed more intensive and extensive gray matter changes than the GE‐GTCS: with GMV loss and covariance connectivity increment. The findings implicated the brain structural changes in GTCSs were organized with a cortico‐striato‐thalamo‐cerebellar network at a large temporal scale.

We found that both the two types of GTCS presented widespread atrophy in the brain, including cortex, thalamus, striatum (caudate heads) and cerebellum, indicating the underlying pathophysiological substrate that GTCS is a maximal event involving the entire nervous system (Aghakhani et al., 2004; Kay et al., 2013; Moeller et al., 2010). These abnormalities within the multiple structures also motivated us, and laid the foundation to investigate the characteristics network organization in epilepsies. Through the comparison analyses, we found that two seizure types both had wide, but not evenly distributed GMV loses in the cortical structures. In GE‐GTCS, GMV loss predominantly located at the frontal lobes, which was consistent with the previous studies using structural and other imaging modalities (Bernhardt et al., 2009; Blumenfeld et al., 2009; Szabó et al., 2006; Wang et al., 2012). Although all cases were from frontal lobe epilepsy, the FE‐FBTS showed more extensive beyond the frontal lobe, and more intensive GMV lose relative to the GE‐GTCS. Wider involvement of cortical structures in FE‐FBTS has also been reported with fMRI (Hamandi et al., 2006) and single photon emission computed tomography (Blumenfeld et al., 2009). The phenomenon might be associated the pathophysiological feature that GE‐GTCS has constant seizure behaviors and specifically selective pattern of involved regions (Bernhardt et al., 2009; Danielson, Guo, & Blumenfeld, 2011; Kay et al., 2013; Moeller et al., 2010). In contrast, FE‐FBTS suffers from partial onset besides to generalized seizures (Italiano et al., 2014; Zhang et al., 2017), might lead to more severe brain impairment.

We combined winner‐take‐all strategy (Buckner et al., 2011; Ji et al., 2015; Wang et al., 2019; Zhang et al., 2008) and structural covariance network analysis (Alexander‐Bloch et al., 2013; Evans, 2013; Mechelli et al., 2005), to comprehensively delineate the network patterns among cortical and subcortical structures in two types of GTCS. Three points should be highlighted regarding the novelty of the work. Firstly, in contrast to the previous works using functional domain (Gotman et al., 2005; Ji et al., 2015; Luo et al., 2012), SCN could reflect the brain network organization under long‐term effect of seizure impairments in months to years (Bernhardt et al., 2009; Hong et al., 2019; Liao et al., 2013; Zhang et al., 2017). Secondly, in contrast to network analysis with unitary seeding region (Ameis et al., 2014; Bernhardt et al., 2009; Chou et al., 2015), winner‐take‐all analysis based on cortical parcellations could refine the cortico‐subcortical networks with specific cortical involvement. Thirdly, the cortico‐subcortical networks were investigated at different subcortical levels of striatum, thalamus, and cerebellum. Overall, both patient groups showed increased covariance connectivity of the cortico‐striato‐thalamo‐cerebellar network, predominantly in the covariances associated with thalamus and striatum. The thalamus is the foremost subcortical structure in epilepsy, plays crucial roles in both generalized and partial seizures with reciprocal cortico‐thalamic networks (Ji et al., 2015; Wang et al., 2019; Zhang et al., 2015; Z. Zhang et al., 2017). In GE‐GTCS, it engages in generation of seizure activity (Výtvarová, Mareček, Fousek, Strýček, & Rektor, 2017; Zhang et al., 2015) and is also the target for stimulating therapy (Mahoney et al., 2018). In FE‐FBTS, it engages in propagation and synchronization of seizure activity (Blumenfeld et al., 2009). The striatum is correlated with specific symptoms in seizures in FE‐FBTS (Blumenfeld et al., 2009), and engages in initiation of seizure activity (He et al., 2020; Mahoney et al., 2018). Abnormalities in the thalamus and striatum have been well demonstrated by functional and structural imaging (Dong et al., 2016; Luo et al., 2012; Wang et al., 2012). This study contributed imaging evidence for supporting epilepsy network from aspect of structural covariance connectivity. Furthermore, we found the cortico‐subcortical connectivity changes was modulated by epilepsy duration. The modulation effects were mostly occurred at the subcortico‐cerebellar covariance connectivity. The cerebellum is topologically connected to cerebrum with complex cortico‐cerebellar networks. Although the cerebellum might play essential role in network inhibition mechanism (Danielson et al., 2011) and is an important target for physiological stimulating therapy (Blumenfeld et al., 2009; Mahoney et al., 2018), it has been commonly regarded as a most impaired structures in epilepsy (Aghakhani et al., 2004; Nelissen et al., 2006). The cerebellar might undergo impairment from seizure propagation with crossed cerebellar diaschisis mechanism (Ferilli, Brunetti, Costantini, & Della Marca, 2018), and presents morphometric atrophy and functional deficits in most epilepsy types (Danielson et al., 2011; Szabó et al., 2006; Zhu et al., 2016). These findings indicated the long‐term seizure impairments refigured specific epilepsy network in the brain.

In concordant with the comparing results of GMV, we further found the covariances associated with frontal, motor and somatosensory cortices were prominently changed in the two epilepsies. These cortical regions mainly receive projection from the ventral anterior nucleus of thalamus (Ji et al., 2015; Zhang et al., 2008). The nucleus is also received motor inputs primarily from striatum, and is engaged in focal and generalized epilepsies (Blumenfeld et al., 2009; Dong et al., 2016; Výtvarová et al., 2017). Likewise, the FE‐FBTS patients, with severer GMV loss, also presented more prominent covariance changes. Previous study has demonstrated that seizure propagation in FE‐FBTS resulted in functional deficits from cortex to thalamus, then to striatum, and ended in cerebellum (Blumenfeld et al., 2009). While in GE‐GTCS, the cortex is involved in seizure incidence through a reciprocal cortico‐thalamus network with source at the thalamus and mid brain (Zhu et al., 2016), the coupling of thalamus‐to‐cortex facilitates propagation and maintenance of seizure activity (Nersesyan, Hyder, Rothman, & Blumenfeld, 2004). These might explain the finding that FE‐FBTS had more intensive and extensive imaging changes than GE‐GTCS.

Several limitations in this work should be noted. Firstly, follow‐up data for longitudinal analysis were lacking, which might be helpful for validating the modulating results of disease duration. Secondly, several variables that might contribute to structural alterations in patients were not considered, such as age (Li et al., 2013) and drug therapy (Kay et al., 2013). Thirdly, we only used epilepsy duration for describing the progression of epilepsy, and did not take account into the frequency or total times of seizure incidences (Zhang et al., 2017). Fourthly, the possible effects of partial seizures in FE‐FBTS were not taken as factor for analysis.

## 5 CONCLUSION

In this work, we used a winner‐take‐all strategy‐based structural covariance connectivity analysis, to delineate the cortico‐striato‐thalamo‐cerebellar networks in two syndromes of generalized epilepsy. Our findings implicated cortico‐striato‐thalamo‐cerebellar network changes at a large temporal scale in GTCS, with FE‐FBTS showing more severe network disruption. The study contributes novel imaging evidence for understanding the different epilepsy syndromes associated with generalized seizures.

## CONFLICT OF INTERESTS

The authors declare no conflict of interest.

## Data Availability

The data that support the findings of this study are available from the corresponding author upon reasonable request.
